# Using image analysis as a tool for assessment of prognostic and predictive biomarkers for breast cancer: How reliable is it?

**DOI:** 10.4103/2153-3539.74186

**Published:** 2010-12-23

**Authors:** Mark C. Lloyd, Pushpa Allam-Nandyala, Chetna N. Purohit, Nancy Burke, Domenico Coppola, Marilyn M. Bui

**Affiliations:** 1 Analytic Microscopy Core, Moffitt Cancer Center, Tampa, FL; 2 Department of Pathology and Cell Biology, University of South Florida, Tampa, FL; 3 Department of Graduate Medical Education, Moffitt Cancer Center, Tampa, FL; 4 Department of Anatomic Pathology, Moffitt Cancer Center, Tampa, FL

**Keywords:** Biomarkers, breast cancer, image analysis

## Abstract

**Background:**

Estrogen receptor (ER), progesterone receptor (PR) and human epidermal growth factor receptor-2 (HER2) are important and well-established prognostic and predictive biomarkers for breast cancers and routinely tested on patient’s tumor samples by immunohistochemical (IHC) study. The accuracy of these test results has substantial impact on patient management. A critical factor that contributes to the result is the interpretation (scoring) of IHC. This study investigates how computerized image analysis can play a role in a reliable scoring, and identifies potential pitfalls with common methods.

**Materials and Methods:**

Whole slide images of 33 invasive ductal carcinoma (IDC) (10 ER and 23 HER2) were scored by pathologist under the light microscope and confirmed by another pathologist. The HER2 results were additionally confirmed by fluorescence *in situ* hybridization (FISH). The scoring criteria were adherent to the guidelines recommended by the American Society of Clinical Oncology/College of American Pathologists. Whole slide stains were then scored by commercially available image analysis algorithms from Definiens (Munich, Germany) and Aperio Technologies (Vista, CA, USA). Each algorithm was modified specifically for each marker and tissue. The results were compared with the semi-quantitative manual scoring, which was considered the gold standard in this study.

**Results:**

For HER2 positive group, each algorithm scored 23/23 cases within the range established by the pathologist. For ER, both algorithms scored 10/10 cases within range. The performance of each algorithm varies somewhat from the percentage of staining as compared to the pathologist’s reading.

**Conclusions:**

Commercially available computerized image analysis can be useful in the evaluation of ER and HER2 IHC results. In order to achieve accurate results either manual pathologist region selection is necessary, or an automated region selection tool must be employed. Specificity can also be gained when strict quality assurance by a pathologist is invested. Quality assurance of image analysis by pathologists is always warranted. Automated image analysis should only be used as adjunct to pathologist’s evaluation.

## INTRODUCTION

Approximately two-third of breast cancers are influenced by activation of estrogen receptor (ER) pathway and expressing ER and/or progesterone receptor (PR) in the tumor nuclei. ER/PR testing identifies breast cancer patients who are candidates for hormonal therapy, which can substantially improve their survival.[1,2] Human epidermal growth factor receptor-2 (HER2) protein is overexpressed and HER2 gene is amplified in greater than 20% of the invasive breast cancers. HER2 positive breast cancers have poor clinical outcomes. Since the development of trastuzumab, FDA approved novel human monoclonal antibody that targeted HER2 protein; HER2 testing is used to identify breast cancer patients who can benefit from trastuzumab alone or added to chemotherapy.[3]

Immunohistochemistry (IHC) is an established and routine assay to determine ER/PR and HER2 protein expression using formalin-fixed and paraffin-embedded breast cancer specimens. However, reported up to 20% of IHC results may not be accurate due to various methods and issues related to pre-analytic, analytic and post-analytic aspects.[4] Given the significant clinical benefits of hormonal and targeted therapy and the impact of accurate test results of ER/PR and HER2 in breast cancer, recently, the American Society of Clinical Oncology (ASCO) and the College of American Pathologists (CAP) collaborated to standardize the practice with the goal of controlling variations in testing and interpretation of ER/PR and HER2.[4,5] ASCO/CAP guidelines state that image analysis is a desirable method of qualifying percentage of tumor cells that are immunoreactive for ER/PR and it can be an effective tool for achieving consistent interpretation for HER2 with confirmation from a pathologist. However, the issue of digital analysis for ER/PR and HER2 is not addressed in detail. The literature of digital analysis in ER/PR and HER2 is limited.[6–9]

The objective of this study is: (1) to assess the accuracy and reliability of two image analysis systems, the Aperio ScanScope XD (Aperio, Vista, CA) and Definiens (Munich Germany) Tissue Studio v.1.2, and directly compare the results with semi-quantitative scores of ER and HER2 scored by the pathologists; (2) work with each commercial entity to develop an algorithm for digital analysis to produce most accurate and reproducible research grade results; (3) to identify potential pitfalls in both methods and (4) to generate preliminary data for future studies of tumor heterogeneity and microenvironment of breast cancer using image analysis.

## MATERIALS AND METHODS

This study was reviewed and approved by the Institutional Review Board (IRB) at the University of South Florida.

### Slide Preparation

Thirty three (33) cases of invasive ductal and lobular breast cancer specimens subjected to ER and HER2 IHC studies on representative tumor sections were retrospectively and randomly retrieved from the file of Anatomic Pathology Department of Moffitt Cancer Center from May to August 2009 to include 10 ER and 23 HER2 cases with range from negative to positive scores. Although each patient’s tumor had concurrent ER, PR and HER2 testing, for this study, specimens were randomly selected and not limited to the same patients. Because ER and PR have the same nuclear staining pattern and HER2 has distinct membranous staining pattern, PR was not included in this study. All samples were formalin-fixed and paraffin-embedded tumor slides of biopsy and surgical resections, which were fixed in neutral-buffered formalin between 6 and 48 hours, followed by the processing and embedding guidelines recommended by ASCO/CAP. Freshly (less than 48 h) cut 5 *µ*m unstained slide were subjected for IHC stains using Ventana (Tucson, AZ) BenchMark XT automated slide stainer. For ER staining, after 60-min cell conditioning solution CC1 antigen retrieval, SP1 rabbit monoclonal CONFIRM^™^ anti-ER 1°Ab (Ventana) was incubated for 32 min, followed by UltraVIEW^™^ DAB Detection kit (Ventana). For HER2, after 30-min cell conditioning solution CC1 antigen retrieval, 4B5 rabbit monoclonal PATHWAY® anti-HER2/neu 1°Ab (Ventana) was incubated for 32 min, followed by UltraVIEW^™^ DAB Detection kit (Ventana). The immunostains were done with appropriate controls. The tissue diagnosis and the quality of the immunohistochemical stains were verified by the study pathologists. The HER2 group was also confirmed by fluorescence *in situ* hybridization (FISH) results. Positive controls for HER2 and ER were all performed on known positive breast cancer cases, and negative controls were run for each specimen using negative serum.

### Semi-quantitative Scoring by Pathologists

For ER, the percent of nuclear reactivity was quantified by the number of positive nuclei as a percentage of total tumor nuclear count and the staining intensity was scored as negative (0), low (1+), moderate (2+) and strong positive (3+) by mean nuclear stain density (0–255 dynamic range) for each tumor. For HER2, the percentage of membranous reactivity, completeness of membranous reactivity and staining intensity was recorded. Following guidelines recommended by ASCO/CAP, the criteria for positive ER is that ≥1% of invasive tumor cell nuclei are immunoreactive; negative ER is that <1% of invasive tumor cell nuclei are immunoreactive of any intensity. For HER2, positive is 3+ cell surface protein expression (defined as uniform intense membrane staining) in >30% of invasive tumor cells. Equivocal is 2+ surface protein expression (complete membrane staining either weak or non-uniform but with obvious circumferential distribution in 10% of invasive tumor cells) or 3+ surface protein expression in less than 30% of invasive tumor cells. Negative is 0 or 1+ surface protein expression (defined as no staining or weak, incomplete membrane staining) in any % of invasive tumor cells. Each of the slides was scanned and the tumor areas were selected by a pathologist. At least ten representative tumor areas were scored and both the average intensity and extent of staining were recorded. Three pathologists participated to the manual scoring and an average score was calculated for each case.

### Slide Scanning

Stained whole slide tissue sections were digitally imaged using the Aperio ScanScope XT (Aperio Technologies, Vista, CA) in their entirety (up to 23.4 × 50mm) using a 20 × 0.75NA PlanApo objective for complete 200× magnification using a Basler tri-linear array camera technology (0.50 *µ*m/pixel). Scan time at 20× magnification ranged from 2 to 6 min, depending on the size of the tissue sections. Slide details regarding patient information and pathologist scoring were blinded. Digital images were recorded within the Aperio Spectrum Database to be analyzed. Positive and negative controls, as described above, were scanned and analyzed with each batch of slides.

### Automated Scoring

Quantitative scoring algorithms were customized for each biomarker using commercially available templates from Aperio Technologies and Definiens. For the Aperio product, a combination of the Genie® Histology Pattern Recognition tool was used to select tumor regions of interest, and either the *Membrane Quantification v*_9 cellular analysis tool for HER2 or the *Nuclear Quantification v*_9.1 cellular analysis tool was used. Neither of the In-Vitro Diagnostic (IVD) FDA-approved versions for HER2 or ER was used. Each algorithm was modified to match the requirements of our particular marker and staining set. These adjustments for our nuclear ER stain include segmentation, nuclear curvature threshold, intensity thresholds, size, roundness, compactness and elongation. These modifications for membrane staining include segmentation intensity thresholds, cell size, nuclear size, cell radius, roundness compactness and elongation. For the Definiens TissueStudio^™^ product, the Composer segmentation and classification tool was used to identify the tumor regions of interest, and either the membranous (HER2) or nuclear (ER) attributes of the cells were identified and scored using three multi-parametric (color and size) training algorithm adjustment sliders. Modified settings were optimized for each marker in our image sets.

### Statistical Analysis

The correlation between the pathologist’s semi-quantitative scores and the automated image analysis scoring was evident and therefore complex statistical analysis was not pursued.

## RESULTS

Thirty-three patients with primary invasive ductal and lobular breast cancer with pathology slides stained for HER2 and ER were included in this study to investigate the reliability of automated image analysis tools. Preliminary results indicated that 93.9% (31 of 33, excluding one positive control) of the scores were categorized within the gold standard of the pathologist’s interpretation. Therefore, additional quality control standards were adopted and translated to the algorithms with increase stringency. Final results of the automated image analysis for both the Aperio and Definiens algorithms scored 100% of the 33 cases within range [Tables 1 and 2].

**Table 1:** HER2 data. Pathologist scoring of HER2 stained samples compared with quantitative image analysis

Sample ID	Pathologist		Aperio		Definiens	
	Staining intensity	(%) Staining	Staining intensity	(%) Staining	Staining intensity	(%) Staining
29	1+	5	1+	3.23	1+	0.091
11	1+	15	1+	0.101	1+	0.106
30	0	0	0	<2	0	<2
26	0	0	0	<2	0	<2
10	0	0	0	<2	0	<2
7	0	0	0	<2	0	<2
31	1+	40	1+	68.73	1+	0.073
15	0	0	0	<2	0	<2
19	0	0	0	<2	0	<2
17	1+	<5	1+	3.76	1+	17.27
8	+2	10	2+	10.48	1+	64.98
4	+2	50	2+	15.33	2+	27.30
8	+2	40	1+	0.708	2+	0.147
16	+2	50	2+	11.69	2+	23.15
1	+3	10	3+	22.24	3+	25.07
5	+2	30	2+	18.76	2+	34.76
36	+2	60	2+	12.54	2+	23.41
20	+2	20	2+	9.12	2+	22.20
6	+3	90	3+	73.90	3+	71.87
12	+3	100	3+	56.16	3+	53.29
2	+3	100	3+	77.52	3+	75.99
32	+3	100	3+	78.89	3+	76.20
9	+3	100	3+	76.26	3+	72.78

**Table 2:** ER Data. Pathologist scoring of ER-stained samples compared with quantitative image analysis

Sample ID	Staining intensity		
	Pathologist	Aperio	Definiens
41	3+	3+	3+
54	0	0	0
43	3+	3+	3+
56	3+	3+	3+
46	3+	3+	3+
48	3+	3+	3+
59	1+ to 2+	2+	2+
61	3+	3+	3+
42	2-3+	2+	2+
51	3+	3+	3+

Samples stained for ER were found to be 100% compliant (*n* = 10) within the staining intensity and percentage range set by the pathologists for both the Aperio and Definiens image analysis tools. This was true of 0 (*n* = 1), 1+ (*n* = 1), 2+ (*n* = 1) and 3+ (*n* = 7) cases [Table 2 showing the intensity data].

**Figure 1:**
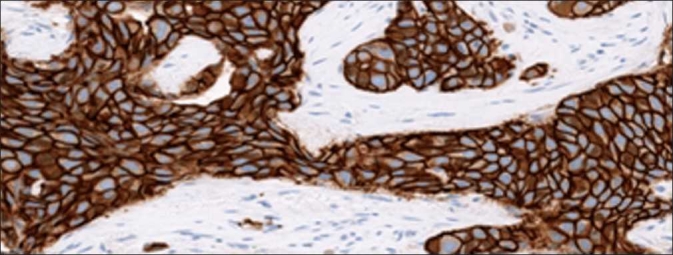
HER2 strong positive percent staining. Pathologist’s score of percent tumor positivity in strongly stained HER2 cases compared directly to quantitative percent positive score results from Aperio and Definiens. An example image of a strong positive sample is included

**Figure 2:**
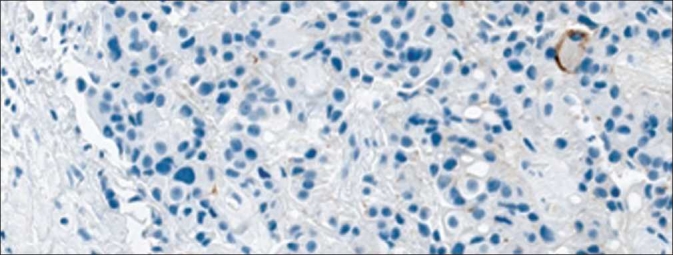
HER2 negative percent staining. Pathologist’s score of percent tumor positivity in negatively stained HER2 cases compared directly to quantitative percent positive score results from Aperio and Definiens. An example image of a HER2 negative sample is included

**Figure 3:**
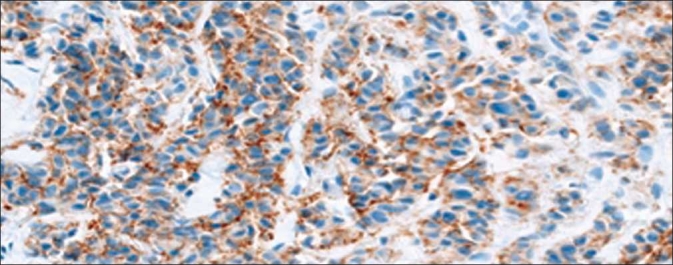
HER2 equivocal percent staining. Pathologist’s score of percent tumor positivity in equivocally stained HER2 cases compared directly to quantitative percent positive score results from Aperio and Definiens. An example image of a equivocally positive sample is included

Samples stained for HER2 were segmented by the pathologists into three categories including: positive, equivocal and negative. The staining intensity and percent positivity were evaluated. Both the Aperio and Definiens derived algorithms scored 5 of 5 positive cases within the intensity range determined by the pathologists, and 10 of 10 negative cases within the identical staining intensity category, as compared to the pathologists. The percent staining for each of these five positive cases was comparable (±3.5%) between each image analysis tool. However, despite being within the established range, the automated percent staining scores were consistently lower than the 90% or 100% estimations given by the pathologists [Figure 1]. In comparison, the automated percent staining scores for the negative group were all within range and 85% within 5% (17 of 20) of the estimates given by the pathologists [Figure 2]. The equivocal cases (*n* = 8) were scored within the appropriate staining intensity ranges, but some discrepancies in percent staining were observed. Each of these samples contained tumors that had HER2 and/or ER positive and negative areas, and various expression levels in the positive areas. These cases were the most challenging for the automated tools to score accurately. For these cases the pathologists scored the percent staining between 10 and 60% whereas the Aperio algorithm scored it within 9.12 and 70.81% and Definiens scored within 14.71 and 64.98% [Figure 3]. These ranges were accepted by the pathologists; however it is critical to note that this variability in scoring is a function of training and classifying objects, as well as of quality control.

## CONCLUSIONS

Historically, ER was measured by a biochemical ligand-banding assay in fresh tumor with well-defined cut-off values of positive and negative results. Currently, the biochemical test has been replaced by the IHC evaluation of formalin-fixed paraffin-embedded tumor tissue sections as a standard of care. The ER IHC test has proven to be highly sensitive, specific and less expensive. The utilization of IHC has several drawbacks or challenges including tumor heterogeneity with staining variability and inter-observer scoring variability.[6] The ER IHC stain has a characteristic nuclear pattern, and it is relatively easy to interpret. Recently, an ER positive tumor has been defined as a tumor having ≥1% invasive tumor cell nuclei.[4] In comparison, HER2 is a membranous stain, which is more subject to inter-observer variability. Although historically the cut-off value for positivity was set as >10% of invasive tumor cells with complete membranous staining, recent recommendation is changed to >30% of invasive tumor cells after correlating with clinical response to trastuzumab.[5]

The introduction of video cameras in the early 1980s which helped to scan the slide has provided new ways of communication and discussion on slides for diagnosis, teaching and research.[10] The first digital scanners were introduced in 2001,[10,11] which combined the advantages of whole slide access with high resolution with digital cameras. It has been shown that the implementation of post-diagnostic scanning for clinicopathological meetings, teaching, consultation and research has overcome many technical challenges and offered many advantages for conferences and consultation.[10] Image analysis of receptor study is another aspect of this advancement and it holds promise for improving inter- and intra-observer reproducibility which is the main pitfall of manual analysis.[12] The drawback though is that no standards of system performance have been yet developed as was for the HER2. Recommendations for standardization are being proposed and include guidelines for sharing image files, transmission of baseline colors, which is of great importance when scoring is based on intensity, as well as a mechanism to evaluate image quality objectively so that they are useful for the pathologists.[11]

With the development of additional new therapies targeted at specific areas of the cancer cell, accurate and reliable measurements are being constantly researched upon for an efficient and reproducible value of these markers. With the advance of technology in all aspects of medicine, the pathology laboratory is of no difference in development and use of technologies to improve its efficiency and result accuracy. Our study indicates that IHC evaluation of receptors in breast carcinoma by image analysis provides data with a substantial agreement with manual scoring. Therefore, image analysis are useful in the evaluation of ER and HER2 IHC stains. Previous studies comparing other image analysis systems with manual scoring reported similar findings.[7,8] Interestingly, the strong concordance of scores was noted to occur when the test areas were selected by the pathologist, or when an automated region selection tool was employed with carefully chosen quality controls. “Teaching” the computer systems to recognize weak brown nuclear staining was inconsistent and had a setback in efficiency early during our evaluation process. However, once the software system was trained, the data analysis concordance with the pathologist scores reached agreement and reduced the rate of false negatives. Usage of the commercially available algorithms was an advantage, which assisted in obtaining reproducibility. Furthermore, FDA-approved algorithms are commercially available to automated image analysis of common biomarkers; however, for research purposes they are extremely limited in the kinds of information which can be reported. Therefore, the modified research grade (non-FDA approved) algorithms were used in order to allow the authors to learn when and how algorithm modification must take place to most efficiently complete high throughput image analysis in a robust and repeatable manner. Finally, adaptable algorithms allow investigators to expand upon the research capabilities of the features extracted from these algorithms for future studies.

Quality assurance by a pathologist is mandatory to assist the instrument in differentiating between benign and malignant cells with accuracy and to verify the scoring by image analysis. Specificity was observed when strict quality assurance by a pathologist was invested. The advantage of using the automated scoring is that, despite being pathologist dependent, it is highly reproducible and it removes the subjectivity associated with manual, visual inspection of the stains. The automated scoring would also be an efficient, faster, precise and accurate method of receptor stain scoring in the hands of a well-trained pathologist. However, without a pathologist’s valuable input, we felt the number of discrepant cases would be significant. This was one of the problems experienced by S Gokhale *et al*, in their study comparing two different image analysis systems.[9] A minor but important advantage to using image analysis would be the quick generation of a report by scanning a barcode instead of the usage of transcriptionist. This provides valuable time for the pathologist that could be spent on corrections, re-dictation or other tasks.

Even though commercially available computerized image analysis can be useful in the evaluation of ER and HER2 IHC results, further investigations of the clinical utility and most importantly standardization of image analyzers, which is yet to be established, are warranted. The ASCO/CAP guidelines recommend: (1) the equipment for image analysis must be calibrated and subjected to regular maintenance and internal quality control evaluation; (2) procedures for image analysis must be validated before implementation for clinical testing. Currently the main advantage appears to be for decreasing the inter-observer variability and for reproducibility values. This study also demonstrates that the quality assurance of image analysis by pathologists is warranted and that image analysis should only be used as an adjunct to the pathologist’s evaluation. As stated above that image analysis has many advantages, and it may be able to replace manual scoring for positive and negative cases, however, for equivocal HER2 cases it is challenging for both manual and image analysis.

This was a core facility initiated pilot study to establish efficacy of this method with a practicing pathologist which studied well-established biomarkers as validation towards investigation of unknown biomarkers. This study explored the utility for high-throughput studies and evaluated the benefits, challenges and limits. The results obtained from this study aid us to establish a structure for stringent quality control at many levels.
